# A simulation study on using ^252^Cf for the treatment of esophagus tumor in human phantom

**DOI:** 10.1186/s40658-020-00341-8

**Published:** 2020-12-03

**Authors:** Fatemeh S. Rasouli, Hasan Bakhshandeh, S. Mohsen Salehkoutahi

**Affiliations:** Department of Physics, K.N. Toosi University of Technology, P.O. Box 15875-4416, Tehran, Iran

**Keywords:** Neutron brachytherapy, Monte Carlo simulation, Esophagus tumor, ^252^Cf source, Dose

## Abstract

Due to the sensitivity of this tissue, and the potential for metastasis of its cancer as well, finding accurate methods to be employed for the treatment of esophagus tumors is of especial interest for the researchers. The present study deals with a Monte Carlo simulation of ^252^Cf neutron brachytherapy for treating these tumors using MCNPX (Version 2.6.0) code. The widely accepted AAPM TG-43 protocol has been used to benchmark the simulated source and to examine the accuracy of the modeling. The MIRD human phantom has been used for dose evaluation in the mentioned tumor and in the surrounding normal tissues as well. To decrease the dose delivered to the healthy tissue, using appropriate shields has been proposed. Through dosimetric calculations for several candidates, Pt-Ir 10% with a thickness of 1 cm has been selected as the optimized shield. The depth-dose results as well as the isodose curves corresponding to the presence of the shielded ^252^Cf neutron source in the center of the simulated tumor offer this source as an appropriate candidate to be used for the treatment of the esophagus tumors and sparing normal tissues. For a suggested clinical condition of positioning the source inside the esophagus, the damage to the first depth in spine can be avoided by managing the treatment time.

## Background

Diseases of the esophagus include motility disorders (achalasia, diffuse spam), hiatal hernias, diverticula, perforation, foreign bodies, chemical burns, gastroesophageal reflux disease (GERD), Barrett’s esophagus, benign tumors, and carcinoma, usually occurring in the fifth or sixth decade of life [1]. Cancer of the esophagus, which has a much higher extent (10 to 100 times higher) in some parts of the world including China and the north of Iran, can be of two categorized types: adenocarcinoma and squamous cell carcinoma. The rate of adenocarcinoma is rapidly increasing in the USA as well as in other western countries. It is found primarily in the distal esophagus and gastroesophageal junction [2]. Risk factors for squamous cell carcinoma of the esophagus include chronic ingestion of hot liquids or foods, nutritional deficiencies, poor oral hygiene, exposure to nitrosamines in the environment or food, cigarette smoking or chronic alcohol exposure, and some esophageal medical conditions such as caustic injury. The risk factor for esophageal cancer includes chronic esophageal irritation. There is an apparent association between GERD and adenocarcinoma of the esophagus. People with Barrett’s esophagus, which is caused by chronic irritation of the mucous membrane due to reflux of gastric and duodenal contents, are more likely to have esophageal cancer.

While detection of esophageal cancer at the early stages can make treatment effective, it is often identified at late stages, making relief of symptoms the only reasonable goal of therapy. Treatment can include surgery, radiotherapy, chemotherapy, or a combination of these modalities, depending on the type of cancer cell, the extent of the disease, and the patient’s condition. A standard treatment plan for a person who is newly diagnosed with esophageal cancer includes the following: preoperative combination chemotherapy and radiation therapy for 4 to 6 weeks, and lastly, surgical resection of the esophagus.

It has been found that locally advanced esophageal cancers are resistant to the conventional X- or gamma-ray radiotherapy with low linear energy transfer (LET). Furthermore, the place of these tumors in the human body has made it easily accessible to use the brachytherapy sources [2]. Neutron brachytherapy (NBT) is a form of high-LET radiotherapy and is expected to be effective in killing the radioresistant esophageal cancer cells. NBT using ^252^Cf sources has been the subject of interest in recent years for treating advanced-stage cancers [3, 4]. It has been shown that NBT is more effective than conventional photon brachytherapy in treating radioresistant tumors such as bulky, late-stage tumors, melanomas, and glioblastomas [5].

Clinical NBT sources are designed in three forms: seed, needle, and applicator tube (AT) [6]. Needle type and seeds are generally used for interstitial soft tissue implants and surface applicators. Owing to the fact that manual loading of radioactive sources, which refers to the insertion of radioactive sealed sources into the patient by a staff member (usually the radiation oncologist), involves radiation protection concerns, the safety guide implementers should be considered. The tube sources containing more than 1 mg of ^252^Cf can produce considerably high neutron flux which is of interest in this treatment method. ^252^Cf remote-afterloading devices with three sources (two ovoids and tandem with initial source strength of 0.4 and 1.3 μg of Cf, respectively) have been adapted for gynecological applications [7]. Due to their compact size, these sources not only can be used for the treatment of esophageal cancers, but also for treating rectum and brain tumors [8].

^252^Cf has a half-life of 2.645 years. While this source emits alpha particles with a probability of 96.9%, they cannot escape from the source capsule arisen from their short range in the medium. The remainder, 3.1% of ^252^Cf, decays through spontaneous fission which each of them produces two or three fission fragments as well as an average of 3.77 neutrons [9]. One microgram of ^252^Cf equals 0.536 mCi and emits 2.31 × 10^6^ n/s. These neutrons provide an energy spectrum that is often modeled as either Maxwellian or Watt fission spectrums, with a peak at about 0.7 MeV and a rapid decrement at both higher and lower energies [10, 11]. ^252^Cf also emits photons and beta particles. Nearly half of the photons emitted by ^252^Cf (each microgram emits 1.32 × 10^7^ photons/s) are prompt fission gamma rays, and the others belong to the delayed gamma rays. Though they are quite different in the spectrum, their mean energy is about 0.8 MeV. While the fission product gamma-ray spectrum peaks near the mean energy, the prompt gamma-ray spectrum has a considerable component above 3 MeV and increases exponentially by the decrement of the energy. The gamma ray associated with alpha-particle decay is negligible (< 0.1%). Because the fission products gradually build up in a sealed ^252^Cf source, it is expected that the absorbed dose of gamma rays compared with those of the neutrons emitted from the source would change in time [8].

Considering the importance of esophageal cancers and the advantages of NBT, the effectiveness of using ^252^Cf sources for the treatment of esophagus tumors needs to be investigated in detail. The accuracy and great flexibility have made Monte Carlo a consistent method for simulating the transport of particles through matter to achieve accurate data for designing a treatment plan and pre-clinical tests. There are several worthwhile studies dealing with Monte Carlo simulation of AT Model ^252^Cf source [4, 9, 12–16]. However, to our knowledge, there is not a study devoted to the investigation of the effect of this source in the treatment of esophagus tumors and the dose delivered to the neighboring organs. In the present study, the Monte Carlo method is used to carry out the dose delivered to the esophagus tumor, and the healthy surrounding organs as well, due to the irradiation of ^252^Cf neutrons implanted in a simulated human phantom. For the evaluation and examination of the accuracy of the simulated source (known as benchmarking), the American Association of Physicists in Medicine (AAPM) Task Group No.43 Report in 1995 (TG-43) [17] is used. In order to reduce the dose to the non-target healthy tissues, various materials and thicknesses are tested to optimize an appropriate shield for this source. For dose evaluations, the MIRD phantom containing various organs is used, which had not been taken into account in the previously related published works [12].

The study has been organized in the following sections: a brief introduction of the ^252^Cf source and detailed description of the model used in the present work, explanations about the human phantom employed in the simulations and the methods for dose evaluation as well, balloon and shield designing, and discussion on the results obtained. The simulations and radiation transport calculations in this study are performed with the MCNPX (Version 2.6.0) [18] Monte Carlo code. The results reported correspond to the adequate number of histories with the relative errors of about 1%.

## Methods

### Source geometry

**Fig. 1:**
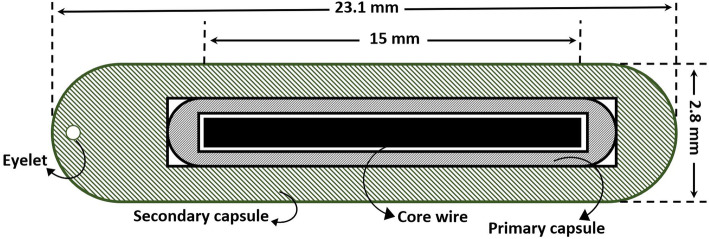
Schematic cross-sectional view of the geometry of AT ^252^Cf source simulated in the present study

In the present work, an AT Model ^252^Cf source, which has been constructed by Oak Ridge National Laboratory (ORNL) [17], was simulated. In this model, the cylindrical active core is Pd: Cf_2_O_3_ ceramic-metal (cermet) with 15.0-mm length and 0.615-mm radius and a mass density of 12.0 g.cm^−3^. The source wire is contained within a primary and secondary capsule of Pt/Ir-10%. A schematic cross-sectional view of the simulated ^252^Cf AT sources is shown in Fig. 1. In our simulations, the mass of the active source is considered to be 32 μg.

The neutron energy spectrum of this source was considered according to the Watt-fission energy spectrum through the following equation:

1 $$ N{(E)}_N={\mathrm{Ce}}^{-0.9756E}\sinh {(2.926E)}^{0.5} $$

where *E* is the neutron energy (in MeV) and *C* stands for a constant factor. The energy spectrum of gamma rays emitted from the source is taken from the study presented by Stoddard and Hootman [19].

### Simulated phantoms

The application of the Monte Carlo method in medical physics covers topics including radiation protection, diagnostic, radiation therapy, and nuclear medicine. Monte Carlo simulation of radiation therapy allows accurate estimation of radiation dose distribution delivered to the patients. As the first step of simulations, the ^252^Cf AT source was located at the center of spherical phantoms containing water, brain, muscle, and A-150 tissue-equivalent plastic. The aim was to estimate the neutron dose rate distribution at a distance of 1 cm from the center of active core, which enables us to compare the results with those reported by Rivard [20]. Material compositions and mass densities, reported in Table 1, have been chosen from report No.44 of the International Commission on Radiation Units and Measurements (ICRU) [21].

**Table 1:** Isotopic composition and mass densities used for water, brain, muscle, and A-150 tissue-equivalent plastic [20]

	Material compositions (weight fraction %)			
Element	Water	Brain	Muscle	A-150
^**1**^**H**	11.19	10.6968	10.1969	10.1297
**C**		0.0032	0.0031	0.0030
^**14**^**N**	-	14.5	14.3	77.5500
^**15**^**N**	-	2.19128	3.38652	3.4918
^**16**^**O**	-	0.00872	0.01348	0.0139
^**17**^**O**	88.81	71.175	70.9716	5.2295
^**19**^**F**		0.0285	0.0284	0.0021
^**23**^**Na**	-	-	-	1.742
^**31**^**P**	-	0.2	0.1	-
^**32**^**S**	-	0.4	0.2	-
**Cl**	-	0.2	0.3	-
**Ar**	-	0.3	0.1	-
**K**	-	0.3	0.4	-
**Ca**	-	-	-	1.8375
**Mass density (g.cm**^**−3**^**)**	1	1.04	1.05	1.127

### Dose evaluation

**Fig. 2:**
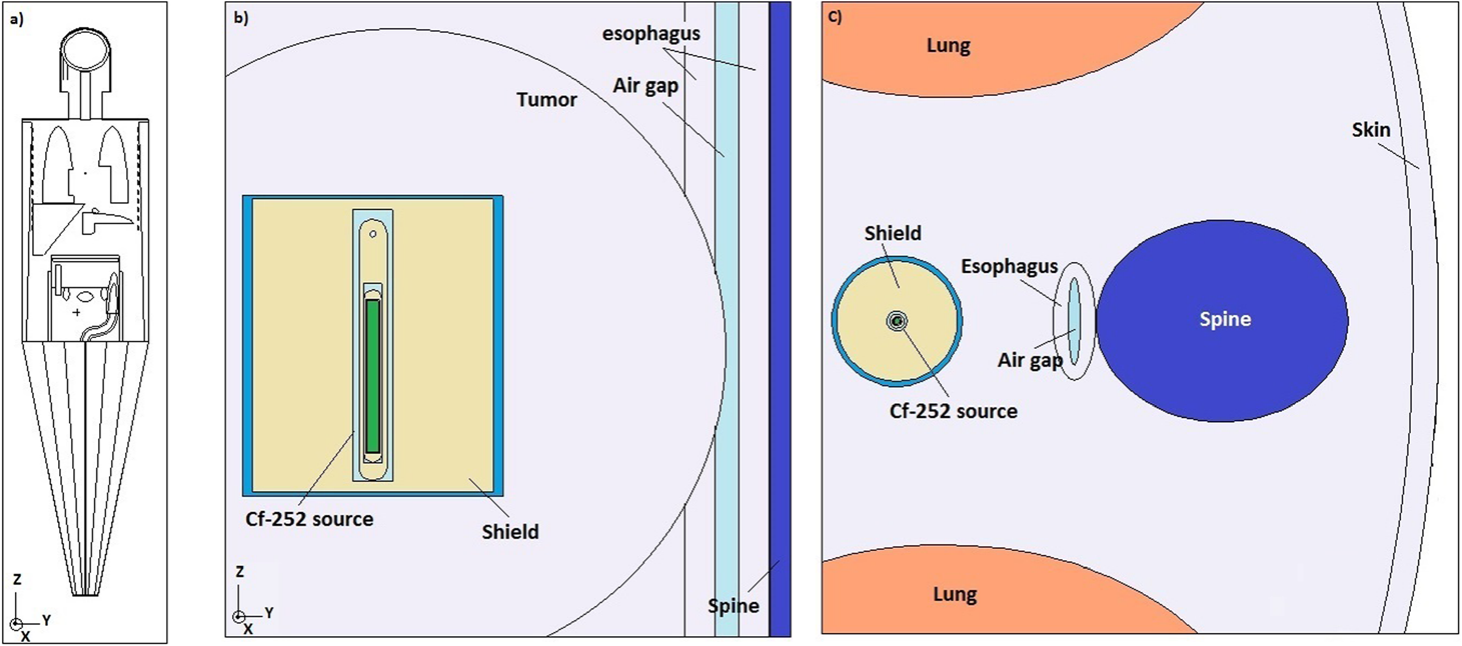
Cross-sectional view of the geometry of **a** simulated MIRD phantom, **b** source and its surrounding shield inside the esophagus tumor, and **c** position of the source in vicinity of the surrounding organs

The MIRD phantom, developed by Fisher and Snyder at ORNL in the 1960s [22], has been used to estimate the dose delivered to the target and surrounding tissues. This phantom includes 35 discreet organs with three materials: soft tissue, bone, and lung. The cross-sectional view of the simulated phantom and the position of the ^252^Cf source in this model is shown in Fig. 2.

The absorbed dose of neutrons in tissue is mainly due to the recoil hydrogen nuclei from elastic scattering interactions. Generally, a neutron transfers approximately half of its kinetic energy to a recoil proton in an elastic scattering interaction. By losing all of its kinetic energy, the neutron is soon captured either by hydrogen via the ^1^H(n,γ)^2^H reaction or by nitrogen via ^14^N(n,p)^14^C reaction. Since the recoiled protons transfer all their energies to the tissue by producing short tracks (< 100 μm) of densely packed ionization events, the absorbed dose of neutrons is referred to as being high-LET. The absorbed dose of gamma rays in tissue is mainly due to the recoiled electrons of Compton scattering interactions. Generally, a photon transfers approximately one-third of its energy to a recoil electron in a Compton scattering interaction. After losing much of its energy in many Compton interactions, the gamma photon is captured through a photoelectric absorption. Because the recoil electrons transfer their kinetic energies in tissue by producing long tracks of sparsely distributed ionization events, the absorbed dose of gamma rays in tissue is referred to as being low-LET.

Generally, one-third of the absorbed radiation dose in tissue near a ^252^Cf source is due to low-LET gamma rays and two-thirds is due to high-LET neutrons. Because the ^252^Cf emission includes both neutrons and gamma rays and owing to that neutrons are more effective than gamma rays (per unit dose) in cell killing, the quantity used for dose prescription is based on Eq. (2):

2 $$ \mathrm{Dose}\left(\mathrm{Gy}-\mathrm{Eq}\right)={\mathrm{RBE}}_{\mathrm{N}}.{\dot{D}}_N+{\dot{D}}_{\gamma } $$

where ${\dot{D}}_N$ and ${\dot{D}}_{\gamma }$ are the neutron and gamma ray dose rates, respectively. RBE_N_ is representative of the relative biological effectiveness of neutrons with respect to gamma rays. This equation implies that the RBE for gamma rays is 1, and clinical results show that RBE_N_ of 6 is an appropriate choice for setting the maximum tolerable dose for normal tissues for many tumor types [23]. A maximum tolerable dose to healthy tissue is supposed to be 12.5 Gy-Eq [24]. The duration of the treatment is reported to be a week and a quarter of this range, which is the time between implanting the source in the patient’s body and taking it away.

## Results

In the first step of simulations, the neutron dose rates were calculated in spherical phantoms of water, brain, muscle, and A-150 plastic. The results are presented in Table 2 and have been compared with those reported by Rivard [20].

**Table 2:** Neutron dose rates at the distance of 1 cm from the center of the phantoms

Phantom	Dose (cGy.μg^−1^.h^−1^)		
	Present work	Rivard [20]	Deviation (%)
**Water**	1.742	1.772	− 1.7
**Brain**	1.694	1.723	− 1.68
**Muscle**	1.630	1.636	− 0.36
**A-150 plastic**	1.684	1.722	− 2.21

**Fig. 3:**
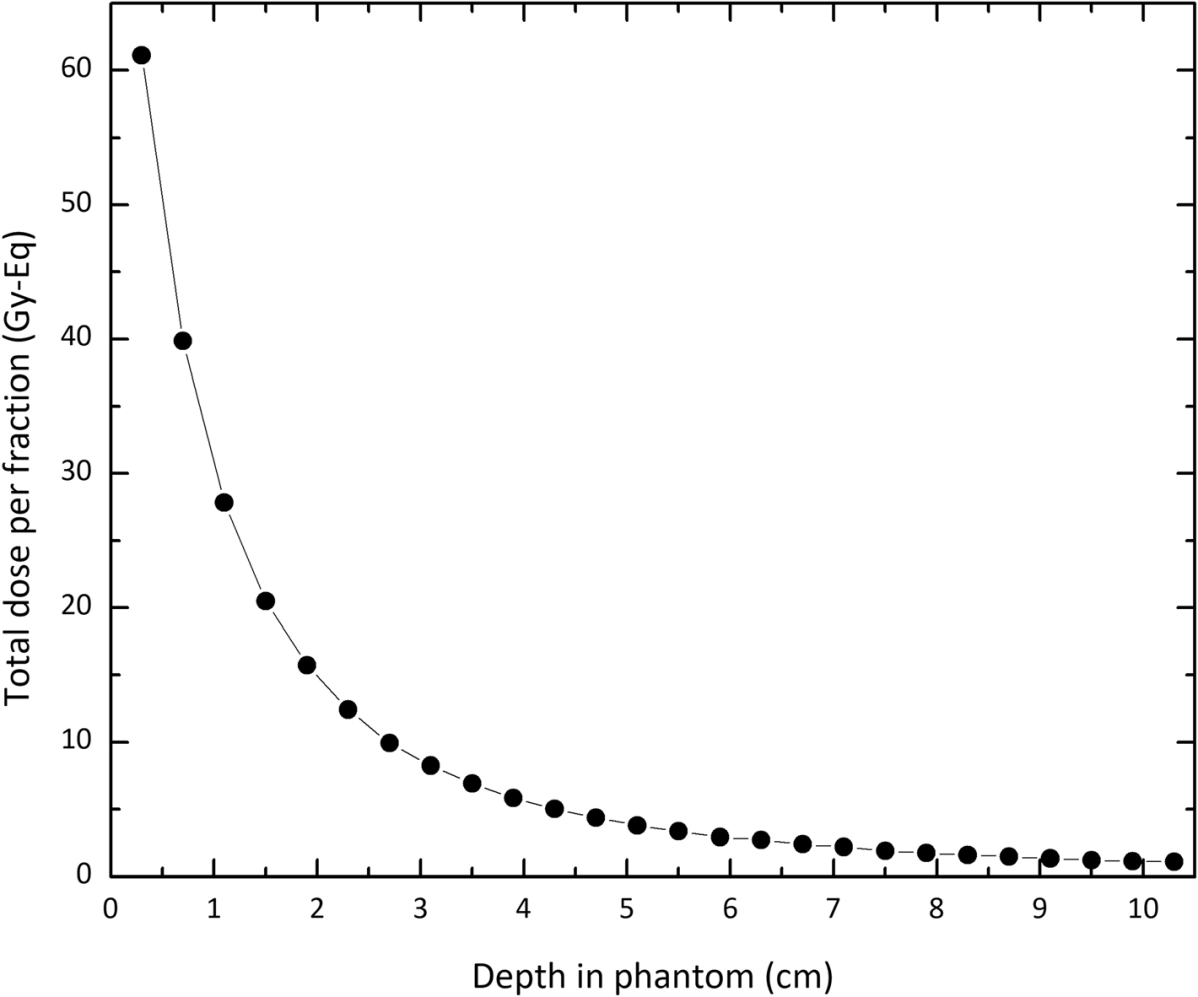
The total equivalent dose in the depth of the phantom (from the surface of the water balloon of 1-cm radius) containing soft tissue. The source has been located in the center of the water balloon

In the experiment, the NBT is used utilizing a water balloon in conjunction with the sources [25–27]. Using this balloon is very important, especially in the case of the tumors located outside the axis of the esophagus. By inflating the balloon in such a condition, its distance can be adjusted close to the tumor and far from the normal tissue [26]. In order to get close to the experimental condition, we have considered this balloon in our simulations: The ^252^Cf AT source was defined at the center of a cylindrical water balloon (2.91-cm high, and 1-cm radius), located in the center of the spherical phantom of 10-cm radius containing soft tissue. Figure 3 shows the total equivalent dose calculated in the depth of the phantom. As the results show, the total dose for depths more than 2.5 cm from the surface of the water balloon (3.8 cm from the source axis) drops below 12.5 Gy-Eq, the tolerable dose for healthy tissue.

**Fig. 4:**
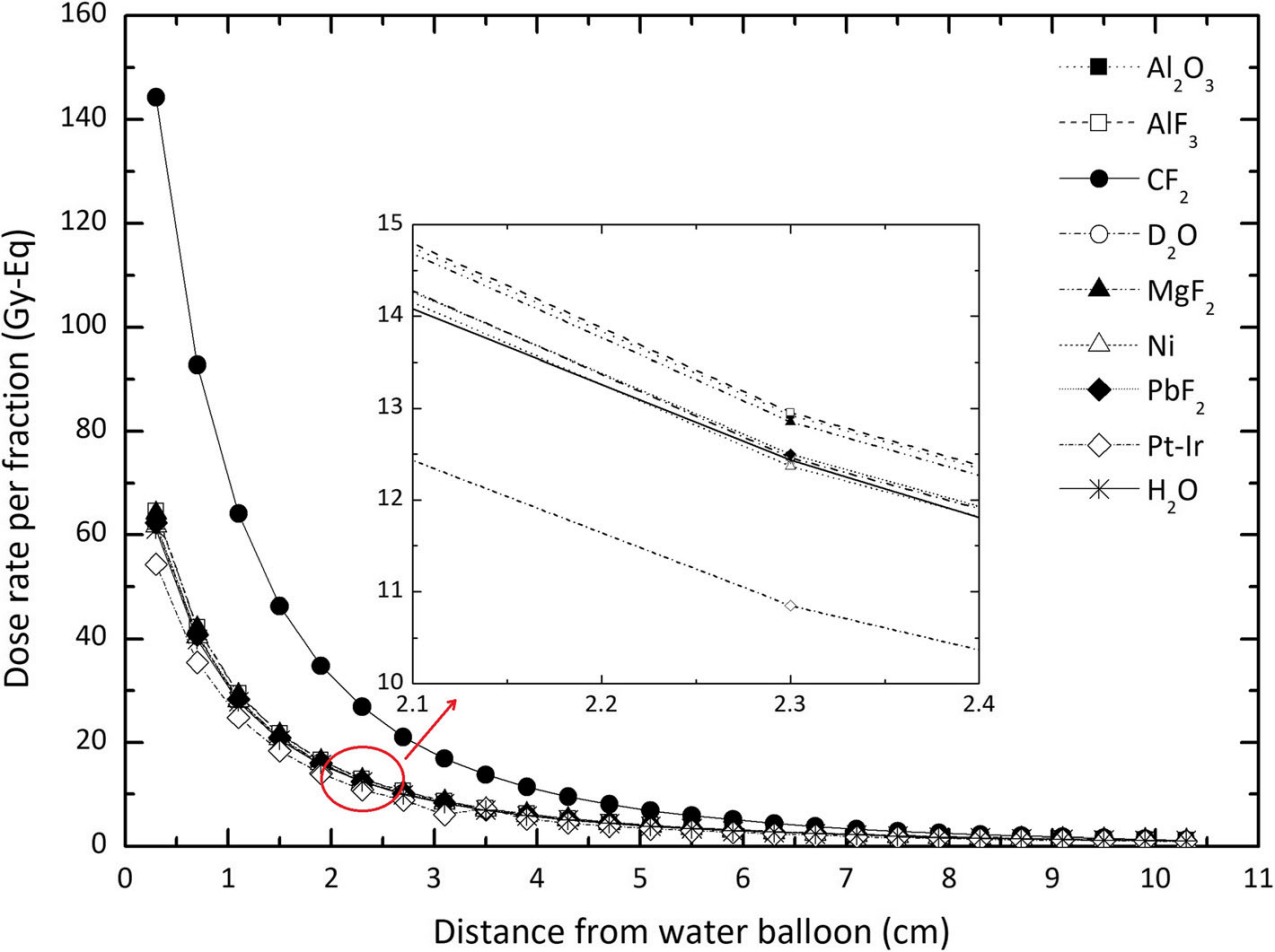
The total dose rates in soft tissue for various distances from the surface of the water balloon containing ^252^Cf source shielded with different materials

In order to satisfy the aim of decreasing damages to the healthy tissues in depths less than 3.8 cm as much as possible, using neutron-absorbing materials as the shield has been proposed. Taking into account the results published so far, nine various materials, including Al_2_O_3_, AlF_3_, CF_2_, MgF_2_, Ni, PbF_2_, Pt-Ir 10%, TiF_3_, and heavy water [28, 29] have been suggested. The shield has been designed as a cylinder of 2.91-cm height and a radius of 1 cm, placed in a water balloon of 1.1-cm radius. According to the results, shown in Fig. 4, Pt-Ir 10% reduces the total equivalent dose in the distance of 2.1 cm from the surface of the water balloon (3.4 cm from the active core) under the allowable limit. According to the data given in Figs. 3 and 4, deviation from the dose delivered to the phantom in the presence of the unshielded source of this quantity for the source encapsulated in Pt-Ir 10% ranges between about 7 and 18%.

**Fig. 5:**
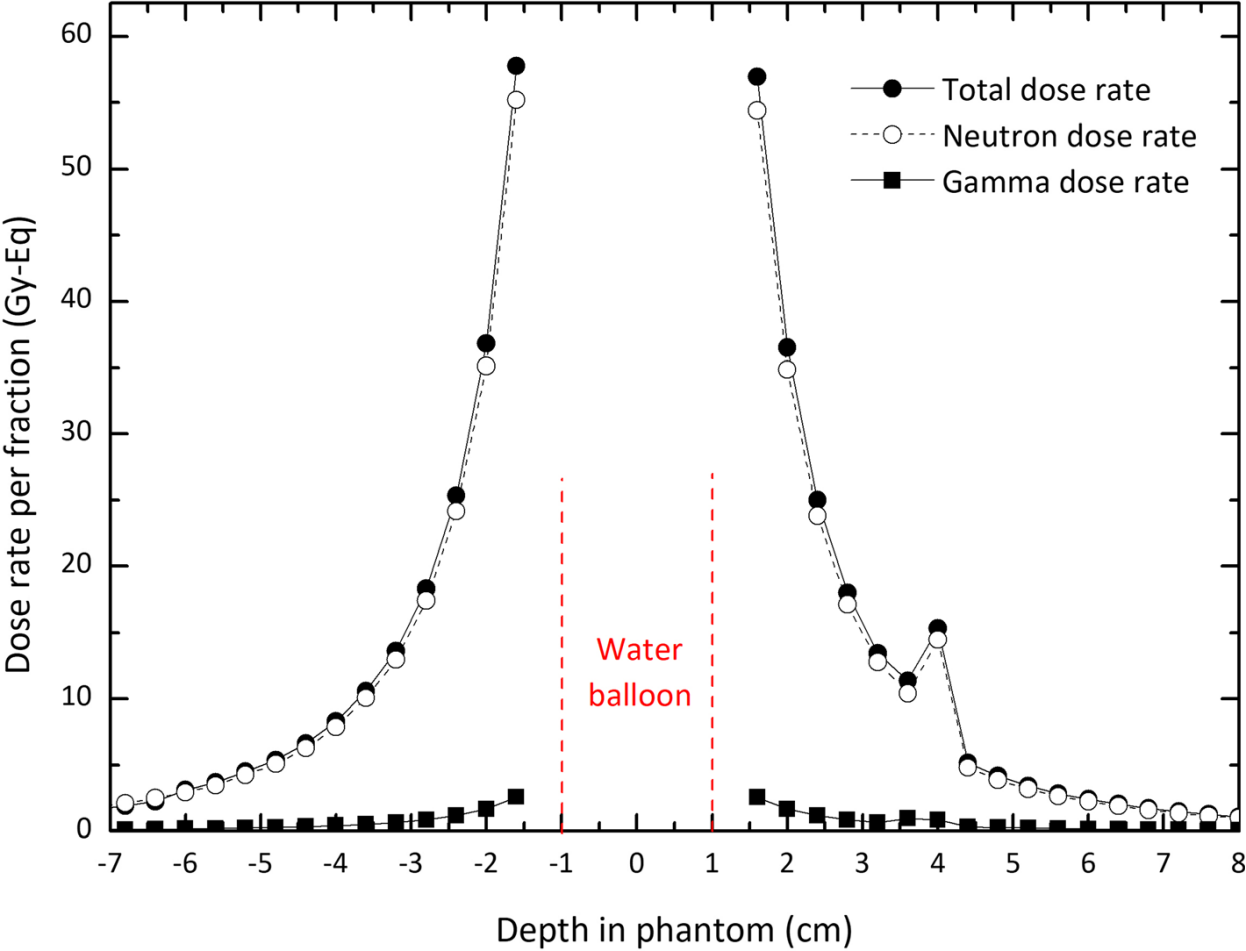
Depth-dose curves for neutrons, photons, and total for two opposite sides of the tumor (see Fig. 2c). The curves in negative and positive depths correspond to the right and left sides of the tumor, respectively

Although these results give an overview of the dose delivered to the tissue due to the presence of ^252^Cf source, the most accurate outlook will be achieved through dosimetric calculations in realistic phantoms. To address this issue, a Monte Carlo-based dosimetry study for ^252^Cf AT source for the treatment of esophageal tumor considering the MIRD phantom has been carried out. In these simulations, the source encapsulated in Pt-Ir 10% (see Fig. 3) has been placed in the center of a cylindrical water balloon of 3-cm height and 1.1-cm radius. The balloon was located in the tumor simulated in the vicinity of the esophagus of the phantom. The depth-dose curves due to the photons and neutrons, and the total dose as well, for two opposite sides of the tumor are presented in Fig. 5. Considering the position of the source within the tumor, the positive depths belong to the right-hand side of the tumor. As the results show, the uniformity in the behavior of dose rate reduction for positive depths has been affected by the presence of different materials (tumor tissue, trachea, esophageal tissue, and spinal cord) in the particles’ path. In this figure, the negative depths belong to the left-hand side of the tumor, which the particles pass through homogeneous soft tissues. This homogeneity has led to the uniform reduction of the dose. According to the dimensions mentioned, the depths between − 1.1 and 1.1 cm correspond to the depths inside the water balloon, and therefore have not been considered in dose calculations. The results indicate that for the depths more than about 3 cm, which is the radius of the simulated tumor, the doses fall to less than the allowable limit of 12.5 Gy-Eq. Strictly speaking, the proposed shield is effective in decreasing damage to the healthy tissue surrounding the tumor.

**Fig. 6:**
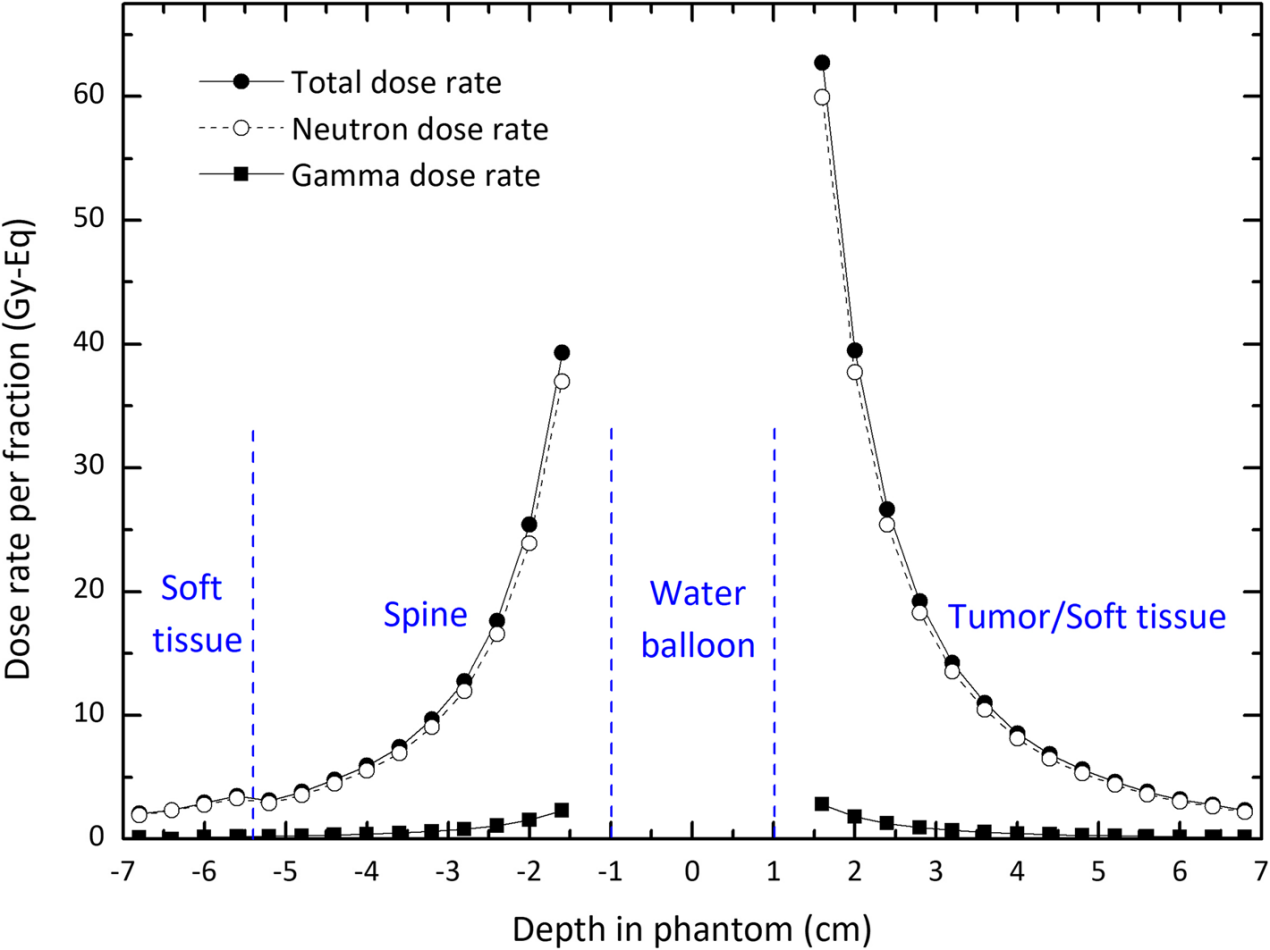
Depth-dose curves for neutrons, photons, and total for two opposite sides of the source located inside the esophagus. The curves in negative and positive depths correspond to the spine and the tumor (and their following soft tissue), respectively. The regions have been shown in the figure

To consider a more realistic clinical condition, the source has been moved within the esophagus. The resultant depth-dose curves are reported in Fig. 6. Considering the position of the source, the positive depths belong to the curves in the tumor and its following soft tissue. The negative depths belong to the data calculated in the spine and the following soft tissue (see Fig. 2). The curves show no significant difference inside the tumor compared to the previous scenario. Due to the higher density, the dose values in the spine decrease by about 60% compared with those inside the tumor. However, the first 1.2 cm depth in this organ exceeds the limit value of 12.5 Gy-Eq. It may be avoided by managing the duration of treatment time.

**Fig. 7:**
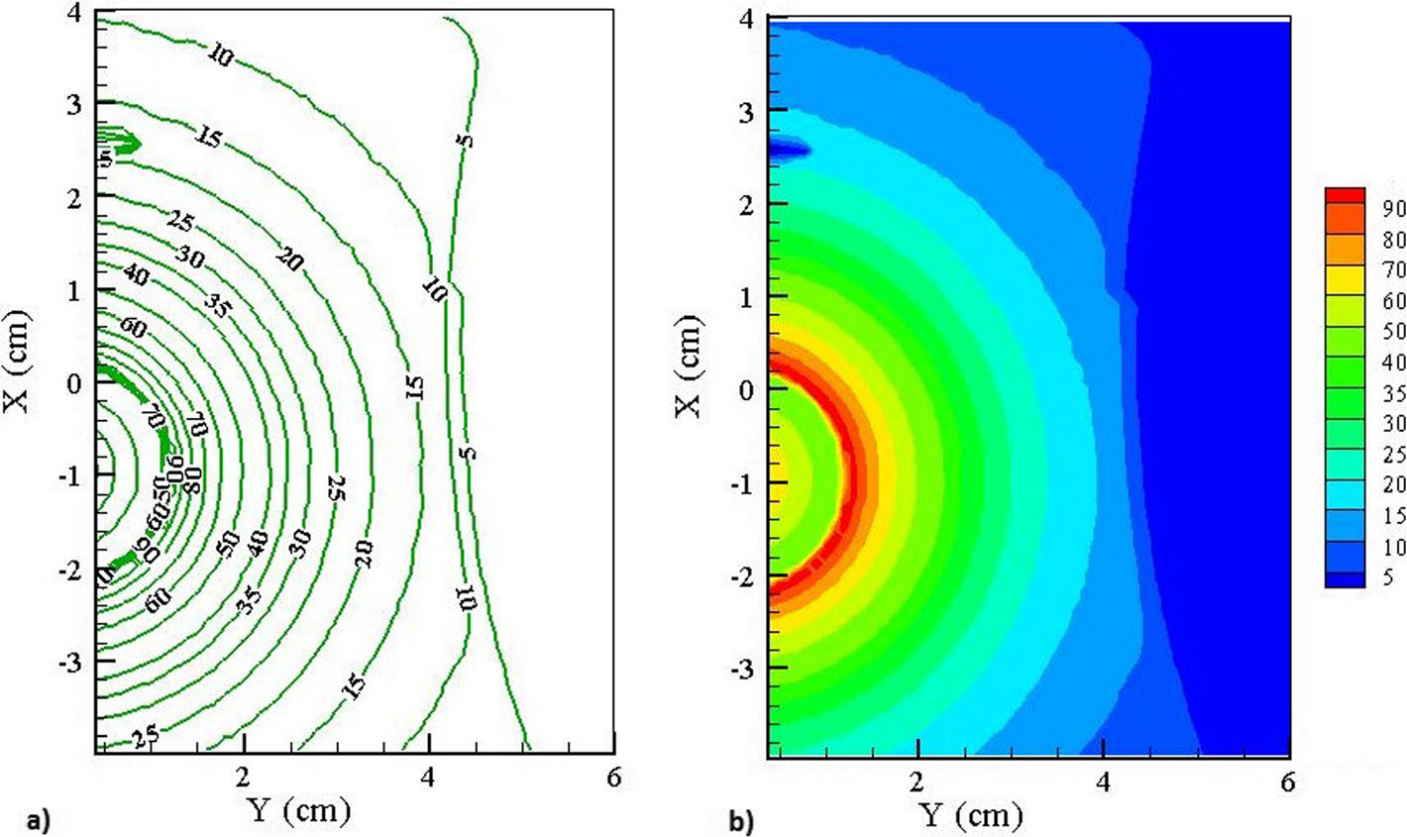
**a** Isodose curves in the esophagus and surrounding tissues. The values are in percent. **b** The color-filled contour plots in the X-Y plane in the esophagus and surrounding tissues. The color scale (in percent) has also been shown

While the depth-dose curves reported in this section give an appropriate prediction of the performance in the direction parallel to the beam axis, the analysis of the dose in various directions will also be of high importance. Therefore, the results have also been plotted in the form of isodose curves to evaluate planar variations in absorbed dose in the phantom. The results corresponding to the plane crossing from the center of the source are presented in Fig. 7a. Also, Fig. 7b presents the color-filled contour plots on this surface in the esophagus and the surrounding tissues. These curves confirm the considerable reduction of the dose delivered outside of the tumor volume.

## Discussion

According to the results, it can be concluded that the ^252^Cf can be considered as a candidate for brachytherapy of esophagus tumor and surviving surrounding healthy tissue. Though the present study aims to investigate the efficiency of neutrons for delivering the desired dose to the target of interest, employing photon radioactive sources has previously been suggested in other works. For example, Neves et al. [30] have accomplished a simulation-based study for brachytherapy of esophagus tumors using ^192^Ir sources in three different configurations. They have evaluated the dose distribution for a female adult mesh anthropomorphic phantom not only in the tumor but also in several healthy tissues.

Table 3 presents a comparison between the dose (in percent) delivered to some sensitive organs due to the irradiation of ^192^Ir source encapsulated in platinum [30] and our designed ^252^Cf source. According to these data, it can be found that for our proposed shielded source, the esophagus which is located in the vicinity of the tumor receives about 30% of the total dose to the tumor, and the damage to the other investigated organs is predicted to be negligible. The results corresponding to the case in which the source is located inside the esophagus (see Fig. 6) also show the similar behavior in these organs. The results behave similarly to those of the ^192^Ir brachytherapy source. It is worthy to mention that though the human phantom used in our study contains 35 different organs, the materials used for all tissues, except the bone and lung, are considered to be soft tissue. Such simplification can lead to quantitatively different results with those of a human phantom with realistic materials for tissues, albeit still gives an appropriate understanding of the effects of using ^252^Cf source for treatment. Considering a more sophisticated model and extending this study to a phantom containing a greater number of organs and materials offers new ways to accomplish more researches in brachytherapy.

**Table 3:** Dose deposition (in percent) to the esophagus tumor for our work and ^192^Ir brachytherapy source encapsulated in platinum

Tissue	Our results (%)	Neves et al. [30]
Tumor	100	100
Lung	3.3	14.1
Esophagus	30.46	184.2
Brain	0.002	0.6
Liver	0.05	3.1

## Conclusion

This study was devoted to assessing the feasibility of using a ^252^Cf source for brachytherapy of esophagus tumors employing a simulated human phantom containing various organs. The simulated source was benchmarked with the widely accepted AAPM TG-43 protocol, and the accuracy of the simulation was tested through a comparison of the results with those of published works. The results show that the designed source shielded with Pt-Ir 10% has the potential to be employed for delivering the desired dose rate to the tumor while preventing healthy surrounding tissue as much as possible. These doses are mostly due to the neutrons, and the equivalent gamma rays’ dose is negligible compared to those of neutrons.

According to the results, as the depth in the phantom increases, the more the dose delivered decreases. However, in the presence of the shield, the dose outside the tumor will not exceed the allowable limit. Moreover, the treatment can be accomplished in a short duration of the therapeutic period which is one of the most important factors in optimizing the patient’s recovery. The results for a more realistic condition of positioning the source inside the esophagus show that though the dose to the tumor does not affect significantly, the dose delivered to the first 1.2 cm in the spine exceeds the allowable limit for healthy tissues. It may be avoided by decreasing the treatment time in each radiation fraction. However, the more distant organs are not damaged in this position for the source.

## Data Availability

All data generated or analyzed during this study are included in this published article.
